# Medication errors in primary health care records; a cross-sectional study in Southern Sweden

**DOI:** 10.1186/s12875-019-1001-0

**Published:** 2019-07-31

**Authors:** Sofia Säfholm, Åsa Bondesson, Sara Modig

**Affiliations:** 1 Tåbelund Primary Health Care Center, Eslöv, Sweden; 2 Institution for Clinical Sciences in Malmö/Center for Primary Health Care Research, Lund University, Box 50332, SE-202 13 Malmö, Sweden; 3 Department of Medicines Management and Informatics in Skåne County, Kristianstad, Sweden

**Keywords:** Medication discrepancies, Medication errors, Medication reconciliation, Primary health care, Patient safety

## Abstract

**Background:**

Drug-related problems due to medication errors are common and have the potential to cause harm. This study, which was conducted in Swedish primary health care, aimed to assess how well the medication lists in the medical records tally with the medications used by patients and to explore what type of medication errors are present.

**Methods:**

We reviewed the electronic medical records (EMRs) at ten primary health care centers in Skåne county, Sweden. The medication lists in the EMRs were compared with the results of medication reconciliations, which were performed telephonically in a structured manner by a physician, two weeks after a follow-up visit to a general practitioner. Of 76 patients aged ≥18 years, who on a certain day in 2016 were visiting one of the included primary health care centers, a total of 56 were included. Descriptive statistics were used. The chi2-test and the Mann Whitney U-test were used for comparisons. The main outcome measure was the proportion of correctly updated medication lists.

**Results:**

Following a recent visit to the general practitioner, a total of 16% of the medication lists in the medical records were consistent with the patients’ actual medication use. The mean number of medication errors in the medical records was 3.8 (SD 3.8). Incorrect dose was the most common error, followed by additional drugs without indication/documentation. The most common medication group among all errors was analgesics and among dose errors the most common medication group was cardiovascular drugs.

**Conclusion:**

A total of 84% of the medication lists used by the general practitioners in the assessment and follow-up of the patients were not updated; this implies a great safety risk since medication errors are potentially harmful. Ensuring medication reconciliations in daily clinical practice is important for patient safety.

**Electronic supplementary material:**

The online version of this article (10.1186/s12875-019-1001-0) contains supplementary material, which is available to authorized users.

## Background

Drug-related problems (DRPs) due to medication errors are common. According to the World Health Organization (WHO), medication errors are a leading cause of avoidable harm within healthcare and organizational adverse events occur in about one in every ten hospitalizations [1]. A medication error is “a failure in the treatment process that leads to or has the potential to lead to harm to the patient”, with the consequent potential to cause adverse drug reactions [2, 3]. The definition includes addition, withdrawal or changed dosage of drug without documentation. In addition, the broader term medication discrepancies also refers to changes in frequency or formulation of medication [4, 5]. Many medication errors are potentially harmful [6, 7] and are often a result of inadequate communication across the various levels of the healthcare system [8]. Besides the human suffering, this is also costly. However, a large proportion of unplanned drug-related hospitalizations are avoidable [1]. Interventions to prevent misunderstandings related to drug regimen include medication reconciliation and may be carried out by GPs or pharmacists through phone interviews, home visits or face-to-face consultations in the clinic [9].

Medication reconciliation has been acknowledged to be an effective strategy for preventing DRPs [10]; a measure that is also highlighted by the Swedish Association of Local Authorities and Regions, SALAR [11]. A medication reconciliation is “the process of creating the most accurate list possible of all medications a patient is taking — including drug name, dosage, frequency, and route — and comparing that list against the physician’s admission, transfer, and/or discharge orders, with the goal of providing correct medications to the patient at all transition points” [12]. Nevertheless, insufficiently updated medication lists are common. A review of the existing literature has shown that between 20 and 87% of patients encounter medication discrepancies upon discharge from hospital [13] and there was a correlation between the numbers of drugs a patient was on and the number of discrepancies; this correlation was also identified in primary health care [14]. This implies that patients with multi-morbid conditions are at greater risk of medication discrepancies since they often receive more medicines. In Swedish primary health care, Ekedahl et al. showed in 2012 that errors in the pharmaceutical and prescription lists are very common when comparing patient data with medical records and prescription database. Eight out of ten patients had at least one discrepancy between current drug use and the medication list [15].

The physician plays a key role concerning medication safety. The Swedish National Board of Health and Welfare emphasizes the general practitioner’s (GP’s) responsibility in this area as well as the importance of routines and support in primary care in order to maintain good quality in the drug treatment, especially for patients with multiple conditions [16].

Many studies have been performed in the context of admission and discharge from hospital regarding medication errors and discrepancies. However, there is a lack of studies in primary health care that identify medication errors by comparing the medication lists with the patients’ actual use.

## Aims of the study

This study, which was conducted in Swedish primary health care, aimed to assess how well the medication lists in the medical records tally with the medications used by patients, given a recent opportunity for the GP to update the list. The secondary aim was to assess what the type of medication errors were.

## Methods

### Setting and study sample

Ten primary health care centers (PHCs) in Skåne county, Sweden contributed with patients. The PHCs were strategically selected in order to get representativeness regarding size, location and whether the PHC was public or privately run. A total of 16 PHCs were invited to participate in the study, whereof six public and four private centers agreed. All patients aged ≥18 years, who on a certain day in May 2016 were visiting one of the included PHCs for a yearly check-up by a physician, were invited for inclusion. The documentation of the symptoms, the assessment and any actions that were taken at the visit, including any medication changes, was expected to be made in an electronic medical record (EMR). The patients received oral and written information about the study by a receptionist after the visit and had the possibility to refuse contact by the researcher. The physicians at the PHCs were not informed about the ongoing study. We excluded patients with multidose drug dispensing (i.e. machine-dispensed disposable sachets in which medications are packaged according to the time of administration [17]) and those who received medication help from the municipal home care. Those who received help from another person in the household for managing the medications were included.

### Procedure

Medication data were collected by the researcher (a resident physician in family medicine) via telephone interviews with the patients two weeks after the index GP visit. The data collection was performed in a structured predetermined manner (Additional file 1). The structured procedure was in accordance with the procedure of medication reconciliation but without collecting medication lists from other caregivers. No other information sources were used other than the patients and the primary health care records. Thereafter, the actual medication use was compared with the documented medication lists in the EMRs at the PHC (the lists that were used at the visits), to assess consistency and the number and type of any medication errors. No measures were taken if any minor errors were identified, i.e. the patients received standard care. However, the researcher would have acted if an error with high risk of serious harm had been identified.

We used the term medication error for any incorrectness in prescription, irrespective of harm outcome or not. The medication errors were classified as (1) “dose errors”, (2) “additional drugs without documentation” and (3) “omitted drugs”, respectively. A dose error included incorrect dosage, uncertain dose due to two or more prescriptions of the same substance, medications prescribed for regular use that were used “as needed” or the opposite. Incorrect time for intake was defined as medication discrepancy – a broader term that also includes all types of medication errors.

If a dose was uncertain due to two or more prescriptions of the same substance, where one prescription was consistent with the use noted at medication reconciliation, all other prescriptions were classified as dose errors. Over the counter (OTC) drugs with ATC code were defined as medications and the medication errors in this group were included in the analysis.

Medication errors were also sorted according to ATC code, except analgesics, which were raised from the respective ATC group and sorted into a separate group.

### Statistics

Descriptive statistics (frequencies, mean and median values and proportions) were used to describe the patients and the number and type of medication discrepancies in their medical records. The chi2-test was used for comparisons between proportions and the Mann Whitney U-test between mean values. *P*-value < 0.05 was regarded as statistically significant.

## Results

Of 76 patients who were asked to participate, 60 agreed and four were excluded due to multidose drug dispensing, having another person responsible for managing medication or changing their mind regarding participation. In total 56 patients were included in the study, of whom 18 were aged ≥75 and 29 (52%) regularly visited a physician not working at the PHC. Background characteristics for the patients are shown in Table 1.

**Table 1:** Characteristics for included patients

*N* = 56	
Women, n (%)	24 (43)
Median age, years (range)	70.5 (30–84)
Number of medications in the records, median (range)	6.0 (1–22)
Number of medications at medication reconciliation, median (range)	6.0 (1–17)

For nine patients (16%; eight patients aged < 75 and one aged ≥75, *p* = 0.15), the medication list in the records was completely consistent with the medications that the patient was actually using.

**Fig. 1:**
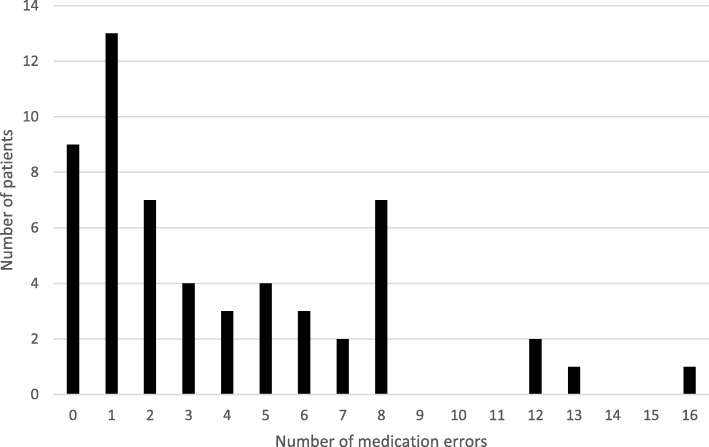
Histogram of the distribution of medication errors

A total of 212 medication errors^1^ were identified. The mean number of medication errors in the medical records was 3.8 (SD3.8) and the median was 2.0 (0–16), reflecting that a few patients showed a lot more medication errors than the main part did (Fig. 1). Among patients who exclusively visited the GP, a mean number of 2.4 (SD3.5) medication errors per list were identified and among those who additionally visited a physician outside the PHC the mean number was 5.0 (SD3.5) (*p* = 0.001).

**Fig. 2:**
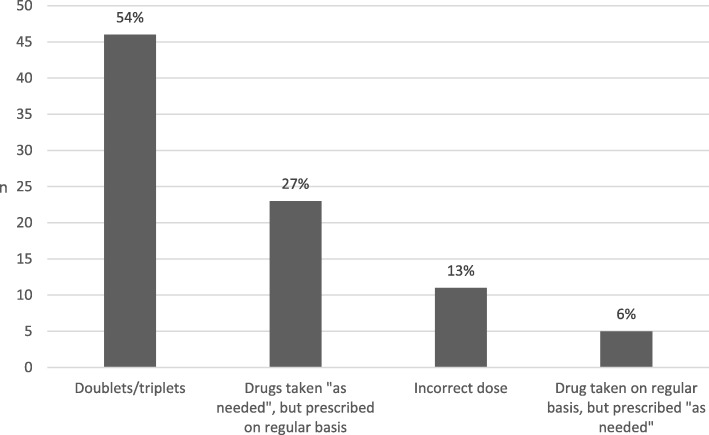
Distribution of dose errors (*N* = 85)

The most common error was dose error. Among all 212 medication errors, 85 were dose errors^2^ (40%). Dose errors were identified among 33 patients (59%). The distribution of dose errors is presented in Fig. 2.

The most common medication group among the medication errors in total was analgesics and for dose errors, cardiovascular drugs were most common.

Out of all medication errors, 80 (38%) were additional drugs, which were present among 27 patients (48%).

A total of 47 omitted drugs were identified; 22% of all medication errors. In total, 23 patients (41%) encountered this type of error. Among patients who only visited a GP, 30% of the lists were lacking one or more medications and among patients who, due to multiple conditions visited another physician outside the PHC regularly (but not subsequently to the index GP visit), this proportion was 52% (*p* = 0.093). No patient had changed his/her drug regimen between the index GP visit and the medication data collection.

In total, four patients used five OTC medications together regularly. None of these medications were included in the medication list in the record (but included in the analysis above regarding omitted drugs). Nine patients used herbal drugs and/or vitamin supplements, most commonly vitamin D.

## Discussion

This study, which was conducted in Swedish primary health care, shows that only 16% of the medication lists in the electronic medical records are consistent with the patients’ actual medication use; a finding that implies that five out of six medication lists used by the general practitioner (GP) in the assessment and follow-up of the patients are incorrect. The medication lists contained on average 3.8 medication errors although a recent follow-up visit to the GP had typically occurred. Incorrect dose was the most common error.

Our findings are in-line with previous research conducted in Sweden and internationally. Ekedahl et al. showed in 2012 that eight out of ten patients in Swedish primary care had at least one medication error between current drug use (assessed by using the Swedish National Drug register [18]) and the medication list [15] and a report from Skåne county in Sweden 2017 stated similar results [19]. What is alarming with the results of the current study is that the errors remained although the GP recently had met the patient for a follow-up visit and thereby had the opportunity to perform a medication reconciliation. Lacking routines for medication reconciliation might be a cause for this and has the potential to result in additional and omitted drugs as well as dose errors. Nevertheless, patient non-adherence or failure to understand the recommended regimen might also contribute to the errors.

International research confirms inadequate medication list updates. A review by Michaelsen et al. showed that between 20 and 87% (median 60%) of patients encounter medication errors upon discharge from hospital [13]. A systematic review from England, which was conducted in 2018, found that medication errors were more likely to occur among older people, or in cases where co-morbidity and polypharmacy was present, i.e. among the most vulnerable patients [20]. Accordingly, in our study, only one medication list out of 18 was consistent with the patient’s actual use for patients aged 75 or more. We also found that the number of medication errors were significantly higher among those patients who, in addition to the GP, regularly also visited another outpatient clinic.

Insufficiently updated medication lists are problematic seeing that many errors are leading to drug-related problems, including adverse drug events [6, 7, 21, 22], which are also causing unplanned hospital admissions [23–25]. According to a previously mentioned review, prescribing in primary care, where most medicines are prescribed and dispensed, accounts for a third of all potentially significant errors [20]. The proportion of prescription errors in primary health care (defined by using indicators) seems to be relatively low; a prevalence of 4.1% of all prescriptions [26]. However, the studies referred to did not compare to actual use, confirmed by medication reconciliations. Furthermore, given the large amount of prescriptions in primary health care, there is still the potential to cause considerable harm in absolute terms. When the severity of medication errors in primary health care was measured, 42% of all errors were described as minor, 54% as moderate and 3.6% as severe [20]. The medication groups that commonly were involved in errors in our study (e.g. analgesics and cardiovascular drugs) are also commonly involved in serious ADEs and are judged clinically relevant according to previous research [21–23].

The most common error described in the literature is omission of medication [13]. In our study, this error was common among those patients who had multiple care providers, i.e. in addition to the GP they also visited another outpatient clinic. This fact further illustrates the importance of medication reconciliation since the GP has the overall responsibility and the medication list in the primary care EMR should optimally be updated and used as the “gold standard”. However, in this study, incorrect dosage was the most common error, most likely due to our choice to classify doublets or several prescriptions of the same drug as a dose error. Dose-related adverse reactions are a common cause of drug-related admissions to hospital [24]. However, Beckett et al. did not find any association between increased risk of harm and a specific type of medication error [22]. The medication list should advantageously be printed out from the record and be given to the patient in relation to every visit in primary health care, not least to prevent usage of the lists “My saved medical prescriptions” at the pharmacy, which might contain several error sources, including doublets with different doses. The risk is impending that the patient takes both tablet strengths and thereby a too high dose. The problem remains if the lists in the records are incorrect.

Keeping the medication lists updated is a basic responsibility in the role as a physician and an accurate medication list is essential to assess the patient’s symptoms as well as the risks and effects of treatment. The Swedish National Board of Health and Welfare especially emphasizes the responsibility of the GP [16] and in 2018 the regulations for drug handling and documentation were tightened [27]. Even so, our study implies that the advice and regulations are not followed. This seems to be particularly true when patients additionally visit other physicians than the GP. Since so few lists are correct, one can also assume that only a few patients received an updated printed medication list during the visit; another aspect where medication safety was lacking. Qualitative research has found variation among GPs concerning understanding about who is responsible for the patient’s medication list and how physicians use different strategies to manage this responsibility [28]. This diversity must be addressed and corrected. Effective performance of reconciliations requires education of physicians and other caregivers. Pharmacist led medication reconciliations prior to physician visits can be effective at reducing the frequency of medication errors in the EMR [29]. However, the access to pharmacists at PHCs in Sweden is low and the intervention must be continuous to maintain an updated list. Opportunities for integrating medication reconciliation with the EMR would facilitate this process. Furthermore, promoting a culture emphasizing medication accuracy is a major patient safety aspect that must be prioritized by health care providers [30].

This study has its strengths and weaknesses. Medication data refers to stated intake from prescriptions and OTC using the procedure of medication reconciliations. This is a strength compared to studies with data collected from the Swedish Prescribed Drug Register [18] and compared to those that identified medication errors by using validated indicators. A standardized questionnaire was used for collecting medication data but the procedure in this study did not include collecting medication lists from other caregivers. It was designed for collecting study data, and is therefore not the same medication reconciliation process that is suggested to be incorporated into clinical practice. One weakness with the study is the limited sample size, since a resident physician in family medicine performed the study as a limited research project. However, data were collected from ten different and strategically selected PHCs in order to get representativeness regarding size, location and whether the PHC was public or privately run. On the other hand, another weakness is that PHCs with staff shortages among physicians and medical secretaries might have declined inclusion; a fact that might affect the results. Furthermore, the physicians who met the included patients were not informed of the ongoing study. Hence we do not know if any of them conducted medication reconciliation at the index visit. However, all patients denied changes of the medication regimen in the two weeks after the appointment.

Future research should focus on how policies regarding medication lists could be efficiently implemented. Furthermore, it would be of value to investigate why physicians do not work sufficiently with medication reconciliations; for this issue qualitative methods would be suitable. As cited by Duguid, “The process of medication reconciliation can significantly decrease errors and is an important element of patient safety.” [31]. Therefore, this method should advantageously be used.

## Conclusion

A total of 84% of the medication lists used by the GP in the assessment and follow-up of the patients were not updated. Medication reconciliations are important for patient safety and must be implemented as a regular routine in daily clinical practice since such an approach can significantly decrease potentially harmful errors.

## Additional file

Additional file 1: The procedure of medication data collection – flow chart. (DOCX 15 kb)

## Data Availability

Data will not be shared, since this was not included in the informed consent from the patients.

## Abbreviations

ADE: Adverse drug event
ATC: Anatomical therapeutic chemical classification
DRP: Drug-related problem
EMR: Electronic medical record
GP: General practitioner
OTC: Over the counter
PHC: Primary care health center

## Footnotes

1 One dose discrepancy regarding incorrect time of dosage was identified, which means that in total 213 medication discrepancies were identified, but 212 medication errors.

2 One dose discrepancy regarding incorrect time of dosage was identified, which means that in total 86 dose discrepancies were identified, but 85 dose errors.
