# Supplementary material for: Medication errors in primary health care records; a cross-sectional study in Southern Sweden
Source: BMC Fam Pract. 2019 Jul 31;20:110. doi: 10.1186/s12875-019-1001-0 (PMC6668157; doi:10.1186/s12875-019-1001-0)
Supplement: Supplementary file 1 — The procedure of medication data collection – flow chart. (DOCX 15 kb) [file 12875_2019_1001_MOESM1_ESM.docx]

**The procedure of medication data collection – flow chart**

First, ask the patient to retrieve his/her medications.

1. Do you manage your medications yourself? If not, who will help you?

2. Do you visit a doctor other than your doctor at the PHC? If yes, who?

3. Review all of the patient's medications based on the patient's own story.
Strength, number of doses /inhalations /injections /applications /suppositories? When does the patient take them?
Note in the current drug list.
If the dose matches the EMR list - note.
Unless – ask if the dose has recently been changed, and by whom?

4. If the patient uses medications that are not in the EMR – note. Ask who the prescriber is (or OTC).

5. If the patient does not mention a medication that is on the list - ask about it. Does the patient take it?

If yes: strength, number of doses /inhalations /injections /applications /suppositories? When does the patient take it? If dose matches the EMR list - note it. If not, ask if the dose has recently changed and by whom?

If no: Stopped treatment? When and by whom? If yes - remove it from the EMR list and record "finished" and by whom (the doctor, the patient himself ...).

6. Capture any more medications by asking if the patient takes medications for:

Pain

Sleep

Heart

Blood pressure

Blood lipids

Stomach

Diabetes

Osteoporosis

Anxiety/depression

Skin

Injections

Inhalations

Eye drops
OTC drugs
Herbal drugs

7. Do you use any medications “as needed”?
Strength, number of doses /inhalations /injections /applications /suppositories? When does the patient use this medication?
If not in the EMR list – note.
If in the EMR list and dose correct – note.
If dose is incorrect – ask if it recently has been changed and by whom? Note.
